# TAE226, a Bis-Anilino Pyrimidine Compound, Inhibits the EGFR-Mutant Kinase Including T790M Mutant to Show Anti-Tumor Effect on *EGFR*-Mutant Non-Small Cell Lung Cancer Cells

**DOI:** 10.1371/journal.pone.0129838

**Published:** 2015-06-19

**Authors:** Hiroki Otani, Hiromasa Yamamoto, Munenori Takaoka, Masakiyo Sakaguchi, Junichi Soh, Masaru Jida, Tsuyoshi Ueno, Takafumi Kubo, Hiroaki Asano, Kazunori Tsukuda, Katsuyuki Kiura, Shinji Hatakeyama, Eiji Kawahara, Yoshio Naomoto, Shinichiro Miyoshi, Shinichi Toyooka

**Affiliations:** 1 Department of Thoracic, Breast and Endocrinological Surgery, Okayama University Graduate School of Medicine, Dentistry and Pharmaceutical Sciences, Okayama 700–8558, Japan; 2 Department of Clinical Genomic Medicine, Okayama University Graduate School of Medicine, Dentistry and Pharmaceutical Sciences, Okayama 700–8558, Japan; 3 Department of Cell Biology, Okayama University Graduate School of Medicine, Dentistry and Pharmaceutical Sciences, Okayama 700–8558, Japan; 4 Department of Hematology, Oncology and Respiratory Medicine, Okayama University Graduate School of Medicine, Dentistry and Pharmaceutical Sciences, Okayama 700–8558, Japan; 5 Department of General Surgery, Kawasaki Medical School, Okayama 700–8505, Japan; 6 Musculoskeletal Disease Area, Novartis Institutes for BioMedical Research Basel, Novartis Pharma AG, CH-4056, Basel, Switzerland; 7 Department of Medical Science Liaison, Department of Oncology Development/Medical Affairs, Novartis Pharma K.K., Tokyo 106–0031, Japan; University of Parma, ITALY

## Abstract

TAE226, a bis-anilino pyrimidine compound, has been developed as an inhibitor of focal adhesion kinase (FAK) and insulin-like growth factor-I receptor (IGF-IR). In this study, we investigated the effect of TAE226 on non-small-cell lung cancer (NSCLC), especially focusing on the *EGFR* mutational status. TAE226 was more effective against cells with mutant EGFR, including the T790M mutant, than against cells with wild-type one. TAE226 preferentially inhibited phospho-EGFR and its downstream signaling mediators in the cells with mutant EGFR than in those with wild-type one. Phosphorylation of FAK and IGF-IR was not inhibited at the concentration at which the proliferation of *EGFR*-mutant cells was inhibited. Results of the *in vitro* binding assay indicated significant differences in the affinity for TAE226 between the wild-type and L858R (or delE746_A750) mutant, and the reduced affinity of ATP to the L858R (or delE746_A750) mutant resulted in good responsiveness of the L858R (or delE746_A750) mutant cells to TAE226. Of interest, the L858R/T790M or delE746_A750/T790M mutant enhanced the binding affinity for TAE226 compared with the L858R or delE746_A750 mutant, resulting in the effectiveness of TAE226 against T790M mutant cells despite the T790M mutation restoring the ATP affinity for the mutant EGFR close to that for the wild-type. TAE226 also showed higher affinity of about 15-fold for the L858R/T790M mutant than for the wild-type one by kinetic interaction analysis. The anti-tumor effect against *EGFR*-mutant tumors including T790M mutation was confirmed in mouse models without any significant toxicity. In summary, we showed that TAE226 inhibited the activation of mutant EGFR and exhibited anti-proliferative activity against NSCLCs carrying *EGFR* mutations, including T790M mutation.

## Introduction

Lung cancer is the leading cause of cancer death worldwide [1]. Lung cancer is divided into two major histological categories, non-small-cell lung cancer (NSCLC) and small-cell lung cancer. Despite great efforts to improve the survival of patients with lung cancer, a satisfactory survival rate has not yet been achieved because the majority of patients present with an advanced disease stage and their tumors exhibit an inherent resistance to conventional chemotherapy [2]. To improve the prognosis of patients with lung cancer, many clinical and basic investigations, including translational research, have been undertaken [3, 4].

Molecular-targeting therapy based on molecular alterations in cancer is a promising strategy for the treatment of lung cancers as well as other malignant tumors. For instance, 4-anilinoquinazoline inhibitors, such as gefitinib and erlotinib, have been designed to inhibit the tyrosine kinase domain of epidermal growth factor receptor (EGFR), which is overexpressed in NSCLC; such inhibitors are currently being used for the treatment of NSCLC [5–8]. Of particular interest, these EGFR tyrosine kinase inhibitors (EGFR-TKIs) produce drastic anti-tumor effects against NSCLCs carrying common *EGFR* mutations, small deletions at exon 19 and L858R mutation at exon 21. Besides these EGFR-TKI-sensitive mutations, T790M mutation at exon 20 is known as EGFR-TKI-resistant mutation [9, 10]. Previous studies have reported that common *EGFR* mutations in the tyrosine kinase domain are more frequent in patients with adenocarcinoma histology, a never smoking history, and an East Asian ethnicity [11–13]. The frequency of common *EGFR* mutation in adenocarcinoma, which depends on the patients’ background, ranges from 15 to 45%, suggesting that the common *EGFR* mutation is a frequent and important event of lung adenocarcinomas [11, 13, 14]. In contrast, inherent T790M mutations are very rare [15]. However, T790M mutations are found in approximately 50% cases among NSCLCs that acquired resistance to EGFR-TKI [16].

Besides EGFR-TKIs targeting EGFR protein, various kinds of small molecule compounds have been developed to target cancer-specific molecular alterations. TAE226, a bis-anilino pyrimidine compound, competes with ATP for focal adhesion kinase (FAK) and insulin-like growth factor-I receptor (IGF-IR). This compound reportedly inhibits cell proliferation, migration, and the invasion of many cancers such as glioma, Barrett’s esophageal adenocarcinoma, ovarian, colorectal and breast cancers [17–22].

FAK is a non-receptor tyrosine kinase that transduces signals from integrin receptor and participates in multiple cell functions required for cell proliferation, survival, motility, and invasion [23]. IGF-IR is a transmembrane receptor tyrosine kinase that participates in signaling cascades that lead to the activation of both AKT and mitogen-activated protein kinase (MAPK) [24]. These tyrosine kinases are known to be overexpressed in many malignant tumors including some NSCLCs and to play an oncogenic role in cancer cells [25–27]. These facts and background encouraged us to examine the anti-tumor effect of TAE226 in NSCLC, and we unexpectedly found that TAE226 preferably inhibits the proliferation of *EGFR*-mutant NSCLC cell lines including the T790M mutant, compared with *EGFR*-wild-type NSCLC cell lines.

In this study, we describe the anti-tumor effect of TAE226 on *EGFR*-mutant cells *in vitro* and *in vivo* and investigated the anti-tumor mechanism of TAE226 in *EGFR*-mutant cells.

## Materials and Methods

### Ethics Statement

This study was carried out in strict accordance with the recommendations in the Guide for the Care and Use of Laboratory Animals of the National Institutes of Health. The protocol was approved by the Animal Care and Use Committee of Okayama University (Permit Number: OKU-2007232). All surgery was performed under ketamine and xylazine anesthesia, and all efforts were made to minimize suffering.

### Reagents

NVP-TAE226 was kindly provided by Novartis Pharma AG (Basel, Switzerland). ZD1839 (gefitinib) was kindly provided by AstraZeneca (Wilmington, DE). The stock solution of these compounds was respectively reconstituted in the concentration of 20 mM and 10 mM with dimethyl sulfoxide (DMSO) (Sigma-Aldrich, St. Louis, MO) and stored at -20°C until used for *in vitro* experiments.

### Antibodies

The primary antibodies to the following proteins were used for western blotting: polyclonal anti-EGFR, phospho-EGFR (Tyr1068), IGF-IRβ, phospho-IGF-IRβ (Tyr1131), AKT, phospho-AKT (Ser473), p44/p42 mitogen-activated protein kinase (MAPK), and phospho-p44/p42 MAPK antibodies were purchased from Cell Signaling Technology (Beverly, MA). Monoclonal anti-focal adhesion kinase (FAK), phospho-FAK (pY397) antibodies were purchased from Biosource (Berkeley, CA), and anti-actin antibody, used as equal loading controls, was purchased from Sigma-Aldrich.

### Cell lines and cell culture

To assess the effect of TAE226, the following 17 NSCLC cell lines, one breast cancer cell line (SK-BR-3), one gastric cancer cell line (MKN45) and HEK-293T cell line were used for *in vitro* and *in vivo* experiments. HCC827, HCC4006, NCI-H3255, NCI-H1975, NCI-H820, NCI-H1819, NCI-H1666, NCI-H1395, NCI-H2228, NCI-H1648, NCI-H1993, NCI-H838 and NCI-H1299 cell lines were kindly provided by Dr. Adi F. Gazdar (University of Texas Southwestern Medical Center at Dallas, Dallas, TX, USA), who established those NSCLC cell lines with Dr. John D. Minna [28–30] except for NCI-H3255, which was established by Dr. Bruce E. Johnson [11, 31]. These cell lines were proven to have individual genetic origins by the Powerplex 1.2 system (Promega, Madison, WI) at University of Texas Southwestern Medical Center at Dallas, before we obtained these cell lines [29]. PC-9 (catalog number: 37012) was purchased from Immuno-Biological Laboratories (Takasaki, Japan). MKN45 (catalog number: JCRB0254) was purchased from Japanese Collection of Research Bioresources Cell Bank, National Institute of Biomedical Innovation (Ibaraki, Japan). SK-BR-3 (catalog number: HTB-30), A549 (catalog number: CCL-185), Calu-3 (catalog number: HTB-55) and HEK-293T (catalog number: CRL-3216) were purchased from the American Type Culture Collection (Manassas, VA). The gefitinib-resistant PC-9 cell line (RPC-9) was established by one of the authors (K.K.) [32]. PC-9, HCC827, HCC4006, NCI-H3255, NCI-H1975, NCI-H820 and RPC-9 have EGFR-TKIs-sensitive mutations. Besides, NCI-H1975, NCI-H820 and RPC-9 cell lines also have T790M EGFR-TKIs-resistant mutation. The details of genetic alterations in these cell lines except for HCC4006 (delL747_A750, P ins) and NCI-H820 (delE746_E749/T790M) are shown in Table 1.

**Table 1:** Characteristics and drug sensitivities of gefitinib and TAE226 in cell lines.

Category	Cell lines	Histological subtypes	Mutational type	IC_50_ (μM) (mean ± SD)	
				TAE226	Gefitinib
*EGFR* mutation	PC-9	AD	Exon 19 del (E746-A750)	0.16 ± 0.01	0.0028 ± 0.0008
	HCC827	AD	Exon 19 del (E746-A750)	0.086 ± 0.01	0.0014 ± 0.00005
	NCI-H3255	AD	Exon 21 (L858R)	0.12 ± 0.005	0.0017 ± 0.0002
	RPC-9	AD	Exon 19 del (E746-A750) + T790M	0.31 ± 0.03	10.8 ± 0.93
	NCI-H1975	AD	Exon 21 (L858R) + T790M	0.17 ± 0.02	7.4 ± 0.29
*EML4*-*ALK* fusion	NCI-H2228	AD	Variant 3	0.28 ± 0.001	11.7 ± 0.64
*BRAF* mutation	NCI-H1666	AD	Exon 11 (G466V)	0.42 ± 0.08	11.9 ± 0.80
	NCI-H1395	AD	Exon 11 (G469A)	0.48 ± 0.002	ND
*KRAS* mutation	A549	AD	Codon 12 (G12S)	1.4 ± 0.05	21.6 ± 1.0
*HER2* amplification	NCI-H1648	AD	WT	1.7 ± 0.07	8.7 ± 0.65
	NCI-H1819	AD	WT	3.8 ± 0.4	6.6 ± 0.059
	Calu-3	AD	WT	4.1 ± 0.37	5.3 ± 0.64
	SK-BR-3	BC	WT	35.1 ± 0.53	ND
*MET* amplification	NCI-H1993	AD	WT	0.89 ± 0.001	12.5 ± 0.36
	MKN45	GC	WT	1.10 ± 0.06	ND
No alterations	NCI-H838	AD	WT	6.2 ± 0.02	28.6 ± 0.35
	NCI-H1299	LCC	WT	2.8 ± 0.26	32.6 ± 1.6
Trasfected HEK-293T cell lines	Mutant EGFR (L858R)		Exon 21 (L858R)	0.41 ± 0.06	0.38 ± 0.087
	Wild type EGFR		WT	3.0 ± 0.11	18.2 ± 3.9

IC_50_, inhibitory concentration at 50%; SD, standard deviation; AD, adenocaricinoma; BC, breast cancer; GC, gastric cancer; LCC, large cell carcinoma; del, deletion type mutation; WT, wild type; ND, not done.

NCI-H3255 was grown in ACL-4 medium [33, 34] supplemented with 10% fetal bovine serum (FBS) and the other cancer cell lines were maintained in RPMI-1640 medium (Sigma-Aldrich) supplemented with 10% FBS, 100 U/ml penicillin, and 100 μg/ml streptomycin (Sigma-Aldrich). HEK-293T cell line was maintained in Dulbecco's Modified Eagle's Medium (Sigma-Aldrich) supplemented with 10% FBS. All the cell lines were maintained at 37°C in a fully humidified atmosphere of 5% CO_2_ in air. All *in vitro* experiments were conducted with about 80% confluent cultures.

### The effect of TAE226 on IGF-IR after serum-starvation

To assess the effect of TAE226 on IGF-IR under ligand-free condition, we analyzed the induced IGF-IR phosphorylation after serum-starvation followed by TAE226 treatment. After cell lines were serum-starved for three hours and treated with increased concentration of TAE226 for two hours, they were stimulated by 100 ng/ml IGF-I recombinant (Sigma-Aldrich) for 15 minutes and then were analyzed by western blotting.

### Determining the drug sensitivity to TAE226 and gefitinib in various cell lines

Drug sensitivity was determined by a modified MTS assay with CellTiter 96 AQueous One Solution Reagent (Promega, Madison, WI). Cells were generally seeded on 96-well plates at a density that would yield 80% confluence by the completion of the experiment, varying by each cell line from 3000 to 4000 per well. After that, the medium in the wells was replaced with the medium containing diluted drug solutions of TAE226 or gefitinib in a 4-log range or complete medium, which were distributed in 8-replicate wells. Cells were incubated in the presence of each concentration of TAE226 for 48 hours and of gefitinib for 72 hours at 37°C in a humidified atmosphere of 5% CO_2_ in air. After that, MTS dye was added to each well. The cultures were incubated for another 1 hour at 37°C in a humidified atmosphere with 5% CO_2_. Optical densities of samples were measured at 490 nm using Immuno Mini NJ-2300 (Nalge Nunc International KK, Rochester, NY). Mean optical density at each drug concentration was calculated after discarding the highest and lowest values. The anti-tumor effects of TAE226 and gefitinib for each cell line were shown in terms of inhibitory concentration at 50% (IC_50_), which was determined by plotting the graph of percentage of cell growth inhibition (Y-axis) versus drug concentration (X-axis). IC_50_ values were expressed as mean and standard deviations. The assays were repeated until the standard deviation became less than the mean.

### Western blot analysis

Cells were cultured in 60 mm dishes overnight, and then, treated with DMSO or each concentration of TAE226 and gefitinib. Next, they were washed out with cold PBS and lysed in 1 × cell lysis buffer [20 mM Tris-HCl (pH 7.5), 150 mM NaCl, 1 mM Na_2_EDTA, 1 mM EGTA, 1% Triton, 2.5 mM sodium pyrophosphate, 1 mM beta-glycerophosphate, 1 mM Na_3_VO_4_, 1 μg/ml leupeptin] (Cell signaling Technology) supplemented with Complete, Mini (Roche, Basel, Switzerland) to extract protein. After the protein concentration was measured, equal amounts of total protein (30 μg) were separated by SDS-PAGE and transferred to PVDF membranes. The proteins on membranes were incubated overnight at 4°C with the primary antibodies. The following secondary antibodies were used: goat antirabbit or antimouse IgG-conjugated horseradish peroxidase (HRP) (Santa Cruz Biotechnology, Santa Cruz, CA). To detect specific signals, the membranes were incubated by ECL plus Western Blotting Detection Reagents (Amersham Biosciences UK Limited, Buckinghamshire, UK).

### *In vitro* binding assay of the variant EGFR-TK domains

The binding ability of TAE 226 for variant EGFR-TK domains was examined with *in vitro* binding assay. Variant EGFR-TK domains consisted of wild-type, delE746_A750, L858R, delE746_A750/T790M, and L858R/T790M. The detailed method is described in S1 Method.

### Kinetic interaction analysis of TAE226 and gefitinib against wild-type EGFR and L858R/ T790M EGFR mutant

To characterize the mechanism of the action of TAE226 on EGFR, we performed kinetic interaction analysis of TAE226 and gefitinib (as a control) against wild-type EGFR and L858R/T790M EGFR mutant by Proteros Reporter Displacement Assay (Proteros biostructures GmbH, Munich, Germany), which was used to determine the following parameters: K_d_, *k*_on_, *k*_off_ and residence time. The Proteros Reporter Displacement Assay was performed as described previously [35, 36]. Detailed method was described in S1 Method.

### Animal xenograft mouse model

In this experiment, we used BALB/c-nu/nu female nude mice at 4–6 weeks of age that were purchased from Charles River Laboratories Japan, Inc (Yokohama, Japan). They were maintained under specific-pathogen-free conditions in accordance with the guidelines of the Animal Care and Use Committee at Okayama University. PC-9 and RPC-9 cell lines (4.0×10^6^/100 μl) mixed with 100 μl Matrigel (Corning Life Sciences, Tewksbury, MA) were inoculated subcutaneously into 24 nude mice [4 different groups (n = 6 in each group) by the concentration of TAE226], which had about 20 g body weight. TAE226 was diluted by methylcellulose in the concentration of 0 mg/kg (vehicle), 30 mg/kg, 60 mg/kg and 90 mg/kg. After the tumor volume has reached 200–400 mm^3^, TAE226 was orally administered to each nude mouse once a day for serial 14 days. Every three days, body weight of the mice and the major axis and minor axis of each tumor were measured and calculated tumor volume using the following formula; (major axis) × (minor axis)^2^ × 1/2 [37, 38]. For the 14 days, tumor volume, tumor growth rate and the body weight were analyzed as an average of the each group.

### Statistical analysis

Student’s *t* test was used to compare data between two groups. Data were represented as mean ± standard deviation (SD). *P* < 0.05 was considered as being statistically significant.

## Results

### Anti-proliferative effect of TAE226 on NSCLC cell lines

We evaluated the anti-tumor effect of TAE226 using an MTS assay in 15 NSCLC cell lines, one breast cancer cell line and one gastric cancer cell line (Table 1). Number of MTS assays performed and each IC_50_ value obtained from independent assays are shown in S2 Table. Three NSCLC cell lines (PC-9, HCC827, and NCI-H3255) with only EGFR-TKI-sensitive *EGFR* mutations and two NSCLC cell lines (RPC-9 and NCI-H1975) with both TKI-sensitive and TKI-resistant *EGFR* mutations showed similar IC_50_ values, ranging from 0.086 to 0.31 μM. In contrast, the IC_50_ values for *EGFR* wild-type NSCLC cell lines (n = 10: ranged from 0.28 to 6.2 μM) were higher than those of *EGFR-*mutant NSCLC cell lines although two *BRAF* mutant cell lines and one *EML4*-*ALK* translocated cell line showed intermediate level of IC_50_ values (range, 0.28 to 0.48 μM). These results suggested that TAE226 was more effective in *EGFR*-mutant cell lines, regardless of the presence of the TKI-resistant *EGFR* T790M mutation, than in *EGFR* wild-type cell lines, especially which did not have *BRAF* or *EML4*-*ALK* alterations.

We also examined the anti-tumor effect of gefitinib against 14 NSCLC cell lines (Table 1). Three NSCLC cell lines harboring only EGFR-TKI-sensitive *EGFR* mutation were sensitive to gefitinib (the IC_50_ values were ranged from 0.0014 to 0.0028 μM), while other NSCLC cell lines harboring wild-type *EGFR* (n = 9) or TKI-resistant *EGFR* mutation (n = 2) were resistant (the IC_50_ were ranged from 5.3 to 32.6 μM). These data were consistent with those of previous reports [10, 28].

### Deregulation of FAK, IGF-IR, and EGFR-related proteins after TAE226 treatment in NSCLC cell lines

**Fig 1:**
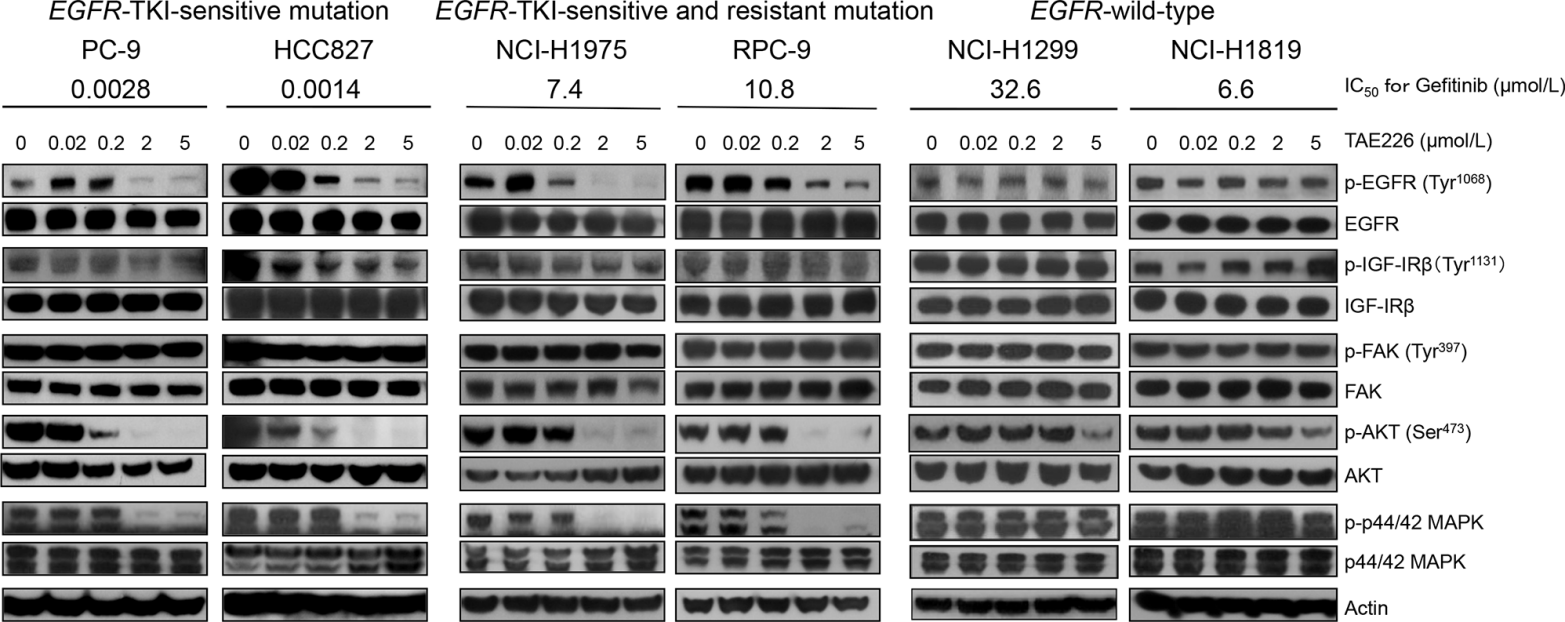
The effects of TAE226 on EGFR-related pathways in NSCLC cell lines. The NSCLC cell lines with EGFR-TKI-sensitive (exon 19 deletions) mutation (PC-9 and HCC827), cell lines with both EGFR-TKI-sensitive and-resistant (T790M) mutations (NCI-H1975 and RPC-9), and cell lines with wild-type EGFR (NCI-H1299 and NCI-H1819) were treated with TAE226 at several concentrations and western blotting analysis was performed. The phosphorylations of FAK and IGF-IR were not suppressed in all NSCLC cell lines tested. In contrast, the phosphorylations of EGFR and its downstream proteins, AKT and MAPK, were suppressed at lower concentration of TAE226 in *EGFR*-mutant cell lines regardless of the presence of T790M mutation compared with *EGFR*-wild-type cell lines. We incubated the membrane for Actin (42 KDa) after we incubated MAPK (42 and 44 KDa) and the stripping buffer was used for that membrane.

We investigated the effect of TAE226 on FAK, IGF-IR, and EGFR-related proteins using 6 cell lines (PC-9, HCC827, NCI-H1975, RPC-9, NCI-H1299, and NCI-H1819). The phosphorylation of FAK (Thy397) and IGF-IR (Tyr1131), which are targets of TAE226, was not significantly inhibited up to a concentration of 5 μM in all 6 cell lines tested (Fig 1). For FAK, phosphorylation was inhibited at a concentration of 20 μM (data not shown). For IGF-IR, phosphorylation was completely inhibited by TAE226 after serum-starvation in all 4 cell lines tested (PC-9, RPC-9, NCI-H1299, and NCI-H1819), regardless of ligand stimulation (S1 Fig). This result indicated that TAE226 inhibited phoshorylation of IGF-IR as expected.

Of particular interest, the phosphorylation of EGFR was predominantly inhibited by TAE226 in all four *EGFR*-mutant cell lines (PC-9, HCC827, NCI-H1975, and RPC-9), regardless of the presence of TKI-resistant mutations. In addition, the phosphorylations of AKT and MAPK, which are downstream molecules of EGFR, were also inhibited in these *EGFR*-mutant cell lines. In contrast, the phosphorylations of EGFR, AKT, and MAPK were not inhibited in cell lines with wild-type *EGFR* (NCI-H1299 and NCI-H1819) (Fig 1). These data suggested that TAE226 might have a stronger effect on mutant EGFR than wild-type EGFR.

We confirmed the expression level of FAK, p-FAK, IGF-IR, and p-IGF-IR in cell lines without drug exposure using ten NSCLC cell lines (*EGFR*-mutant cell lines, PC-9, HCC827, NCI-H3255, HCC4006, NCI-H1975, NCI-H820 and RPC-9; *EGFR* wild-type cell lines, NCI-H1819, NCI-H1299 and A549). No significant difference in the expression level of each of the proteins was observed among the cell lines (S2 Fig).

### Effect of TAE226 on mutant EGFR-transfected HEK-293T cell line

We examined the anti-proliferative effect of TAE226 on two HEK-293T cell lines stably transfected with wild-type EGFR or mutant EGFR (L858R) that we had previously established [39]. The HEK-293T with mutant EGFR was more sensitive to gefitinib than the HEK-293T with wild-type EGFR (the IC_50_ values were 0.38 ± 0.087 and 18.2 ± 3.9 μM, respectively) (Table 1). TAE226 exerted a higher anti-tumor effect on the HEK-293T with the mutant EGFR than on the HEK-293T with the wild-type EGFR (the IC_50_ values were 0.41 ± 0.06 and 3.0 ± 0.11 μM, respectively) (Table 1). TAE226 inhibited the phosphorylations of EGFR and AKT in the HEK-293T cell with the mutant EGFR but not in the HEK-293T cell with the wild-type EGFR (S3 Fig). These results also indicated that TAE226 inhibited the cell growth or survival of *EGFR*-mutant cells by suppressing mutant EGFR protein.

### Binding affinity of TAE226 to mutant EGFR

**Fig 2:**
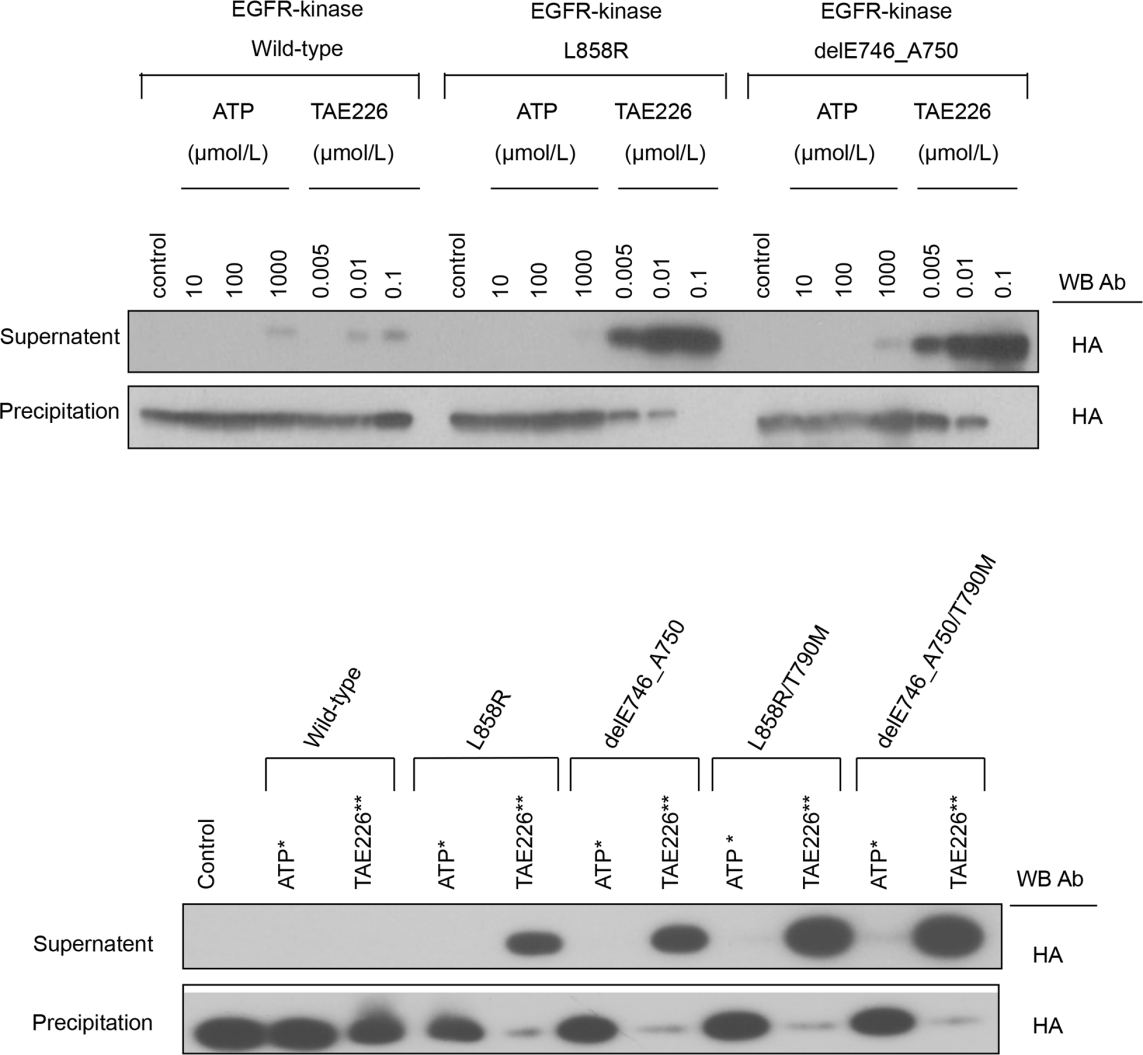
The binding affinity of TAE226 to EGFR kinases. A) common EGFR mutant kinases and B) T790M containing EGFR mutant kinases. The five types of EGFR kinases, wild-type, exon 21 L858R mutation (L858R) or exon 19 deletion (delE746_A750), T790M in tandem with the L858R mutation (L858R/T790M), and T790M in tandem with delE746_A750 (delE746_A750/T790M), which competitively bind to ATP or TAE226, were extracted and were incubated with beads-modified ATP. TAE226 or the recombinant ATP was added to compete with beads-modified ATP for the binding to EGFR kinases. The EGFR kinase binging with the beads-modified ATP was present in precipitation, and the EGFR kinase binding with TAE226 (or recombinant ATP) was present in supernatant. Western blotting was performed to detect EGFR kinases in each component using a HA antibody. *, at the concentration of 10 μM for ATP; **, at the concentration of 0.005 μM for TAE226.

To assess the binding affinity of TAE226 to EGFR kinases, we performed an *in vitro* binding assay of the variant EGFR kinases (Fig 2). The supernatant fraction contained EGFR kinase that did not bind to bead-modified ATP because of interference from TAE226 (or exogenous ATP). The precipitated fraction contained EGFR kinase that had bound to bead-modified ATP, indicating that TAE226 (or exogenous ATP) did not interfere with the ATP binding. Thus, the band intensity at each TAE226 (or exogenous ATP) concentration reflects the ability of TAE226 (or exogenous ATP) to compete with bead-modified ATP for the EGFR kinases.

Among the variant EGFR kinases that were tested (wild-type, L858R, delE746_A750, L858R/T790M and delE746_A750/T790M), no difference in the amounts of EGFR kinases that bound to the bead-modified ATP was noted under the control condition (Fig 2A and 2B). Exogenous ATP weakly bound to EGFR kinases at a concentration of 1000 μM in all the variants tested (Fig 2A). In contrast, TAE226 started to bind to wild-type EGFR kinase at a concentration of 0.1 μM and at lower concentrations for mutant EGFRs, indicating that TAE226 possess a higher affinity to EGFR kinases than ATP. We estimated the binding affinity of TAE226 to EGFR variants by comparing the ratio of the semi-quantified band intensity between the supernatant fraction and the precipitated fraction at 0.1 μM of TAE226 among wild-type, L858R, or delE746_A750 (S4A Fig). Quantification using ImageJ software (National Institutes of Health, Bethesda, MD) showed that the binding affinity of TAE226 was approximately 7.5-fold higher to both mutant EGFRs than to wild-type EGFR (S4A Fig). The effect of acquired T790M mutation on the binding affinity for TAE226 to common mutant EGFR kinases (L858R or delE746_A750) was also evaluated at the concentration of 0.005 μM for TAE226 and 10 μM for ATP (Fig 2B). Interestingly, the binding affinity for TAE226 was higher in T790M containing EGFR mutant kinases (L858R/T790M or delE746_A750/T790M) than in common EGFR mutant kinases (L858R or delE746_A750) (S4B Fig).

### Interaction kinetics of TAE226 and gefitinib with wild-type EGFR and L858R/T790M EGFR mutant

IC_50_ and K_d_ values for TAE226 and gefitinib against wild-type EGFR and L858R/T790M EGFR mutant were shown in Table 2. IC_50_ and K_d_ values for TAE226 were 0.326 μM and 0.163 μM in wild-type EGFR, and 0.0193 μM and 0.00966 μM in L858R/T790M EGFR mutant, respectively. Regarding gefitinib, those were 0.0244 μM and 0.0122 μM in wild-type EGFR, and 0.927 μM and 0.463 μM in L858R/T790M EGFR mutant, respectively. Gefitinib showed a higher affinity of about 40-fold for wild-type EGFR than for L858R/T790M EGFR mutant. On the contrary, TAE226 showed higher affinity of about 15-fold for L858R/T790M EGFR mutant than for wild-type EGFR. The values of *k*_on_ and *k*_off_ could not be calculated in TAE226 for the both type of EGFR and in gefitinib for L858R/T790M EGFR mutant because of the short residence time (< 1.4 min).

**Table 2:** IC_50_ values by kinetic interaction analysis of TAE226 and gefitinib against wild-type EGFR and L858R/T790M EGFR mutant.

Compounds	Purified kinases	IC_50_ (μM) (mean ± SE)	K_d_ (μM)* (mean ± SE)	*k*_on_ (1/s 1/M) (mean ± SE)	*k*_off_ (1/s)** (mean ± SE)	Residence time (min)***
TAE226	wild-type EGFR	0.326 ± 0.0531	0.163 ± 0.0265	NA	NA	< 1.4
	L858R/T790M EGFR	0.0193 ± 0.00129	0.00966 ± 0.000645	NA	NA	< 1.4
Gefitinib	wild-type EGFR	0.0244 ± 0.00175	0.0122 ± 0.000874	369534.789 ± 4941.6	0.00451 ± 0.000383	4
	L858R/T790M EGFR	0.927 ± 0.0983	0.463 ± 0.0492	NA	NA	< 1.4

*K_d_ = 1/2 x IC_50_

**k_off_ = K_d_ x *k*_on_

***residence time = 1/*k*_off_

SE, standard error; NA, too fast to measure; ND, not determined; 1.4 min = shortest residence time that can be measured.

### Anti-tumor effect of TAE226 on NSCLC mouse xenograft models

**Fig 3:**
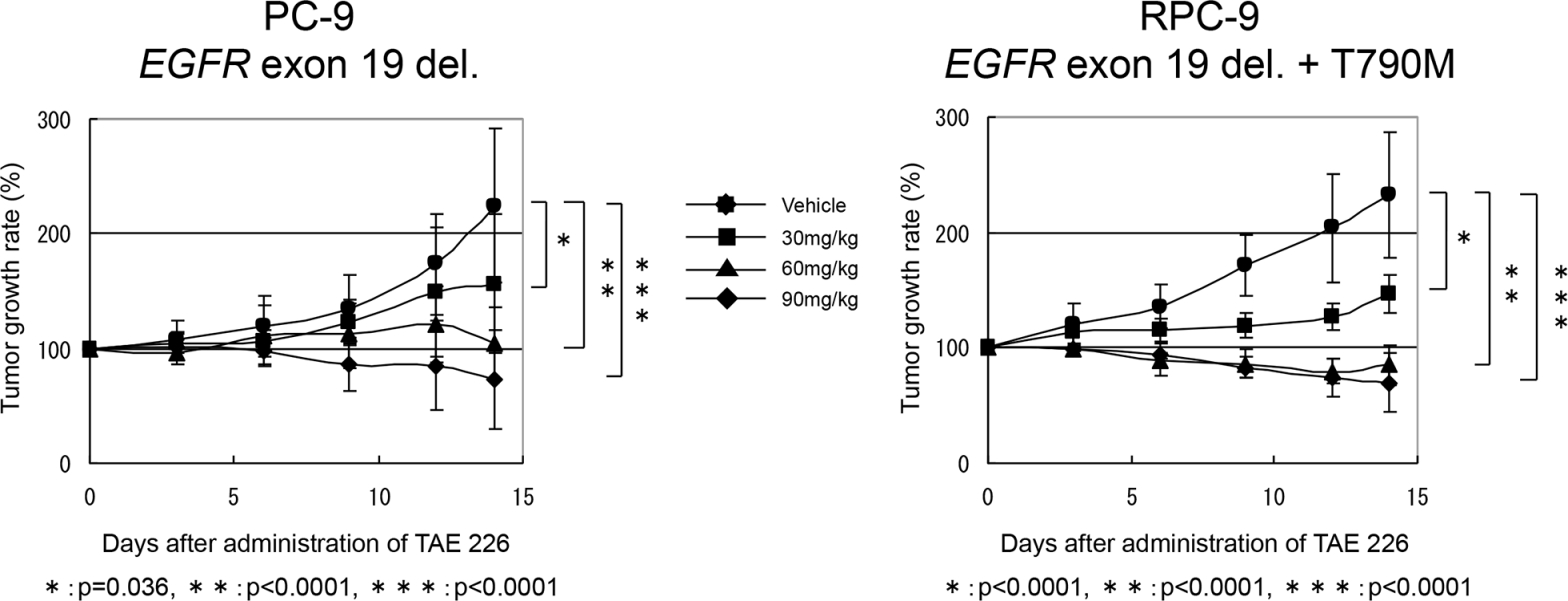
The anti-tumor effect of TAE226 in xenograft models. The oral administration of TAE226 for 14 days significantly inhibited the tumor growth of the subcutaneously inoculated mice xenografts with PC-9 (exon19 deletion) or RPC-9 (exon19 deletion and T790M mutation) at every dose (30, 60 and 90 mg/kg). In addition, TAE226 showed the similar anti-tumor effect on both xenograft models irrespective of the presence of EGFR T790M mutation.

Based on our *in vitro* data, we examined the anti-tumor effect of TAE226 on mouse xenograft models. After the subcutaneous tumor volume of the inoculated PC-9 and RPC-9 cell lines in nude mice had reached a volume of 200–400 mm^3^, we treated the nude mice with the oral administration of TAE226 (30 mg/kg, 60 mg/kg or 90 mg/kg) or methylcellulose as vehicle for 14 days. The tumor volume and body weight of both xenografts were measured on the day before the oral administration of TAE226 and every three days thereafter. Obvious side effects, including the loss of body weight, did not occur in the vehicle-treated group or in the groups treated with 30mg/kg or 60mg/kg of TAE226, while a loss of body weight was observed in mice treated with 90 mg/kg of TAE226. The tumor volume in both xenografts significantly decreased over time and in a dose-dependent manner, compared with the vehicle-treated mice (Fig 3). Furthermore, both xenografts of EGFR-TKI-sensitive (PC-9) and EGFR-TKI-resistant (RPC-9) cell lines showed similar anti-tumor effects in response to TAE226 *in vivo*.

## Discussion

As shown in the relationship between small EGFR-TKI and *EGFR* mutation [40], the ability of TAE226 to compete with ATP, resulting in its anti-tumor potential, is determined by two factors: 1) the binding ability for TAE226 to mutant EGFR and 2) the affinity for ATP to mutant EGFR. It is well known that the affinity for ATP is lower to common EGFR mutants than to wild-type EGFR [40].

There seems to be another important factor that strongly influences the effects of molecular targeting drugs on cancer cells. According to the concept of “oncogene addiction”, *EGFR*-mutant cell lines are highly dependent on mutant EGFR for cell proliferation and survival [41]. Whereas the reason for poor inhibition to FAK or IGF-IR activity in NSCLC is not clear, our findings indicated that TAE226 strongly inhibited the mutant EGFR, compared to FAK and IGF-IR, in cells that are “addicted” to the mutant EGFR, resulting in the anti-tumor activity of TAE226. Of note, there were some discrepancies between IC_50_ of *in vitro* non-cellular kinase assay and cellular inhibition assay for some oncogenes. This phenomenon seems to be explained by the concept of “oncogene addition” that the degree of dependency to specific oncogenes can be different among tumors and oncogenes. IC_50_ values for c-Src (0.91), HER2 (0.95), FGFR-1 (0.75), c-Met (0.16) and ALK (0.15), which are often activated in NSCLC, are less than that for EGFR (1.7) (S1 Table, updated data from ref. 20), indicating that TAE226 may also inhibit these kinases in addition to EGFR in NSCLCs, especially if the tumors are “addicted” to these kinases.

Regarding the mechanism whereby the T790M mutation is thought to influence small molecule TKIs such as gefitinib and erlotinib, the T790M mutation has been thought to cause resistance by sterically blocking the binding of TKIs [9, 10, 42]. In addition, Yun and colleagues reported that an increase in ATP affinity is the primary mechanism by which the T790M mutation causes resistance to TKIs; while the single L858R mutation reduces the binding affinity of ATP by about 30-fold compared with the wild-type EGFR, introduction of the additional T790M mutation restores the binding affinity of ATP comparable to that of the wild-type EGFR [43]. Interestingly, our data showed that IC_50_ values of TAE226 for EGFR T790M in tandem with the L858R (or delE746_A750) mutant cells are similar to those for EGFR L858R (or delE746_A750) mutant cells, providing the contrasting results compared to the IC_50_ values of gefitinib for them. Our *in vitro* binding assay also indicates that the double mutant reveals higher binding affinity to TAE226 than that of the L858R (or delE746_A750) mutant. It is well known and documented that increased hydrophobic interactions between a protein and a ligand lead to an enhanced binding affinity of the ligand, occasionally by several orders of magnitude [44]. Kinetic interaction analysis of TAE226 and gefitinib using wild-type EGFR and L858R/T790M EGFR mutant confirmed that the L858R/T790M double mutant showed higher binding affinity to TAE226 than wild-type EGFR. By taking into account the fact about the binding affinity of ATP to the L858R and L858R/T790M mutants described above, we can reasonably assume that, in the presence of ATP, TAE226 will show comparable inhibition to both mutants, by canceling out the two type of factors–the binding of ATP to the mutants as well as the binding of TAE226 to the mutants. Actually, the IC_50_ of the RPC-9 cells was higher than the IC_50_ of the PC-9 cells for TAE226. However, the difference of IC_50_ between PC-9 and RPC-9 for TAE226 is slight compared to that for gefitinib. As for *in vivo* study, the obtained results suggest the safety of TAE226 doses suitable for the treatment of *EGFR*-mutant cells, regardless of the presence of *EGFR* T790M mutation *in vivo*, opening the possibility for clinical application. In addition to TAE226, related bis-anilino pyrimidine compounds have also been developed [45]. Further study is warranted to examine whether these bis-anilino pyrimidine compounds inhibit mutant EGFR kinase.

In conclusion, we found that TAE226 inhibited the activation of EGFR-mutant kinase and exerted anti-tumor activity on *EGFR*-mutant NSCLC regardless of the presence of T790M mutation. Our results showed that the EGFR L858R/T790M (or delE746_A750/T790M) mutant retains the binding affinity to TAE226 comparable to that of the L858R (or delE746_A750) mutant, suggesting that TAE226, or its relatives, is promising to overcome acquired TKI resistance mediated by *EGFR* T790M mutation.

## Supporting Information

S1 Fig The effect of TAE226 to IGF-IR-related proteins after the stimulation of IGF-I recombinant in NSCLC cell lines. Serum-starved NSCLC cell lines were treated with TAE226 for two hours and stimulated with IGF-I recombinant for 15 minutes. The phosphorylations of IGF-IR and AKT were completely inhibited in all cell lines regardless of *EGFR* mutational status.
(TIF)

S2 Fig The expression profiles of p-FAK, FAK, p-IGF-IR and IGF-IR in NSCLC cell lines. There were no significant differences in the expression levels of p-FAK, FAK, p-IGF-IR and IGF-IR in 10 NSCLC cell lines regardless of *EGFR* mutational status.
(TIF)

S3 Fig The effect of TAE226 on mutant EGFR-transfected HEK-293T cell line. The mutant EGFR-transfected HEK-293T was more sensitive to gefitinib treatment than wild-type EGFR-transfected HEK-293T. TAE226 significantly inhibited phosphorylations of EGFR and AKT in mutant EGFR-trasnsfected HEK-293T compared with wild-type EGFR-transfected HEK-293T.
(TIF)

S4 Fig Comparison of the binding affinity of TAE226 to EGFR variants by the quantification of EGFR protein expression. (A) Density of EGFR protein bands between the supernatant fraction and the precipitated fraction at 0.1 μM of TAE226 in Fig 2A was semi-quantified by densitometry analysis. The binding affinity of TAE226 to EGFR variants was approximately 7.5-fold higher to both mutant EGFRs than to wild-type EGFR. (B) Density of EGFR protein bands in supernatant fraction for TAE226 in Fig 2B was semi-quantified by densitometry analysis. Band density of EGFR protein for TAE226 was higher in T790M-containing EGFR mutant kinases (L858R/T790M or delE746_A750/T790M) than in common EGFR mutant kinases (L858R or delE746_A750).
(TIF)

S1 Method Methods for “*In vitro* binding assay of the variant EGFR-TK domains” and “Kinetic interaction analysis of TAE226 and gefitinib against wild-type EGFR and L858R/ T790M EGFR mutant”. Detailed methods are described.
(DOCX)

S1 Table *In vitro* non-cellular kinase assay for TAE226. This table is updated data from ref. 20.
(DOCX)

S2 Table IC_50_ values in independent assays and calculated SD for TAE226 and Gefitinib in each cell line. Mean IC_50_ and SD were calculated using the value of each IC_50_.
(DOCX)
