# Supplementary figures and images for: TAE226, a Bis-Anilino Pyrimidine Compound, Inhibits the EGFR-Mutant Kinase Including T790M Mutant to Show Anti-Tumor Effect on EGFR-Mutant Non-Small Cell Lung Cancer Cells
Source: PLoS One. 2015 Jun 19;10(6):e0129838. doi: 10.1371/journal.pone.0129838 (PMC4474554; doi:10.1371/journal.pone.0129838)

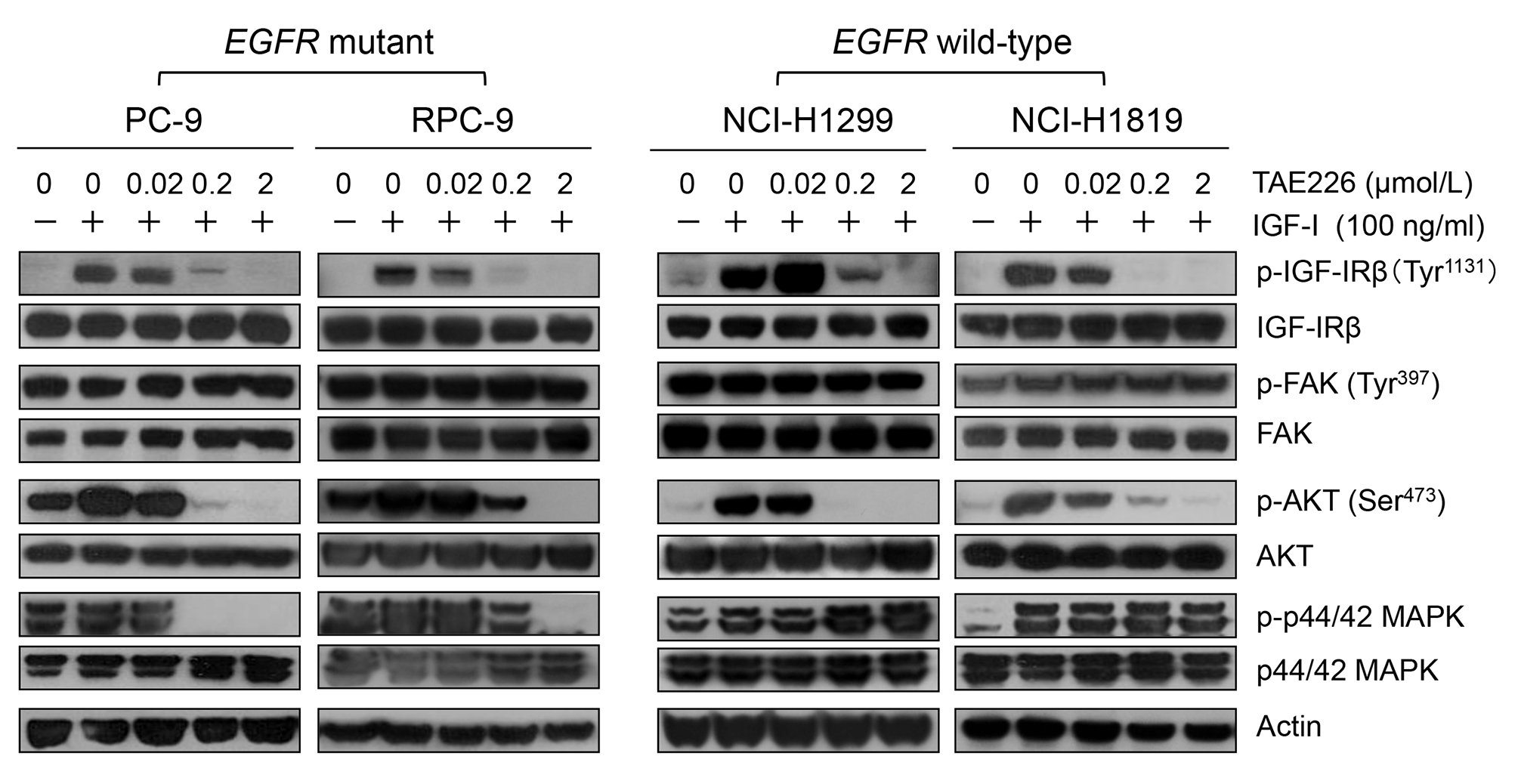

Supplement: S1 Fig — Serum-starved NSCLC cell lines were treated with TAE226 for two hours and stimulated with IGF-I recombinant for 15 minutes. The phosphorylations of IGF-IR and AKT were completely inhibited in all cell lines regardless of EGFR mutational status. (TIF) [file pone.0129838.s001.tif]

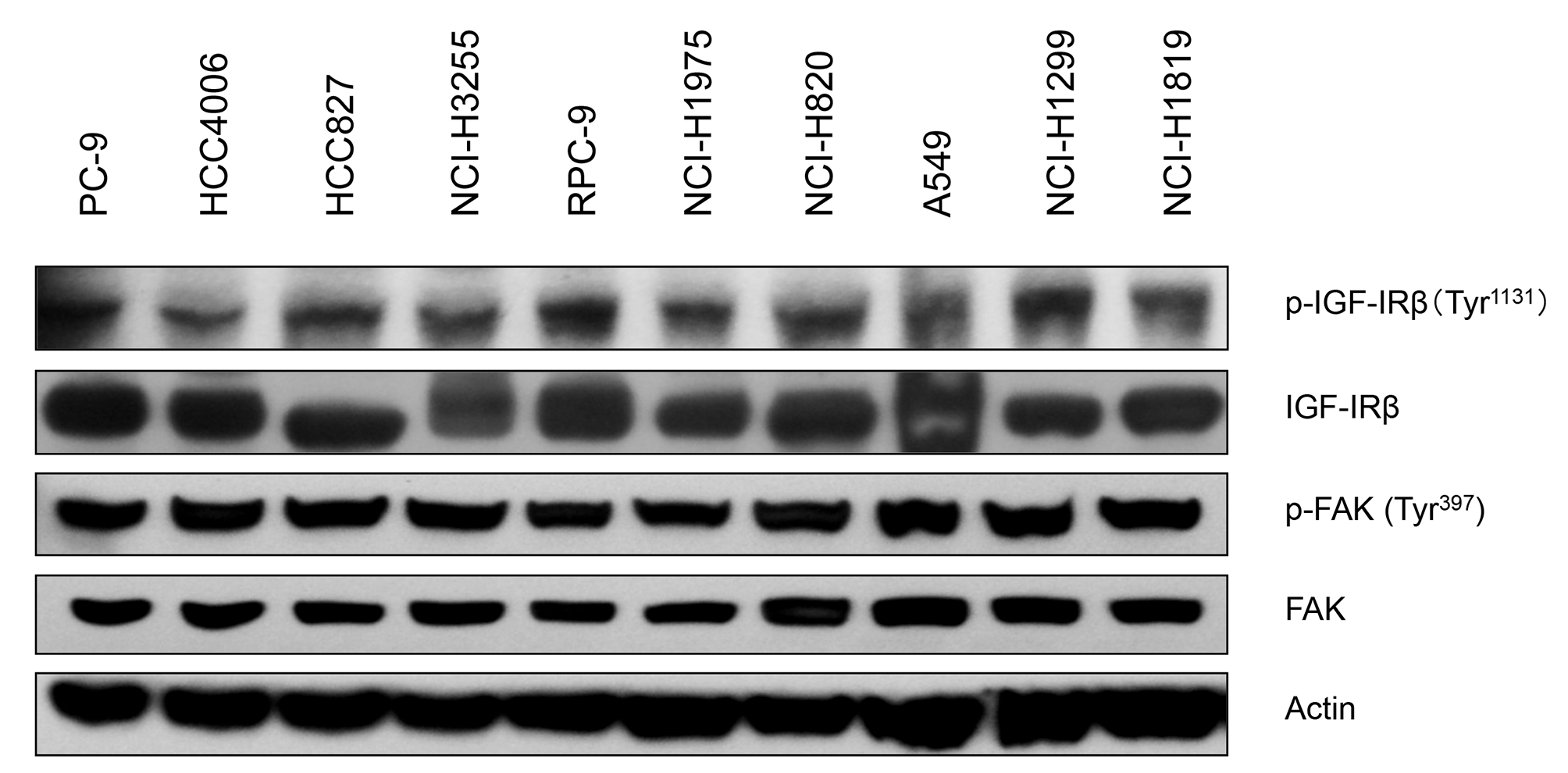

Supplement: S2 Fig — There were no significant differences in the expression levels of p-FAK, FAK, p-IGF-IR and IGF-IR in 10 NSCLC cell lines regardless of EGFR mutational status. (TIF) [file pone.0129838.s002.tif]

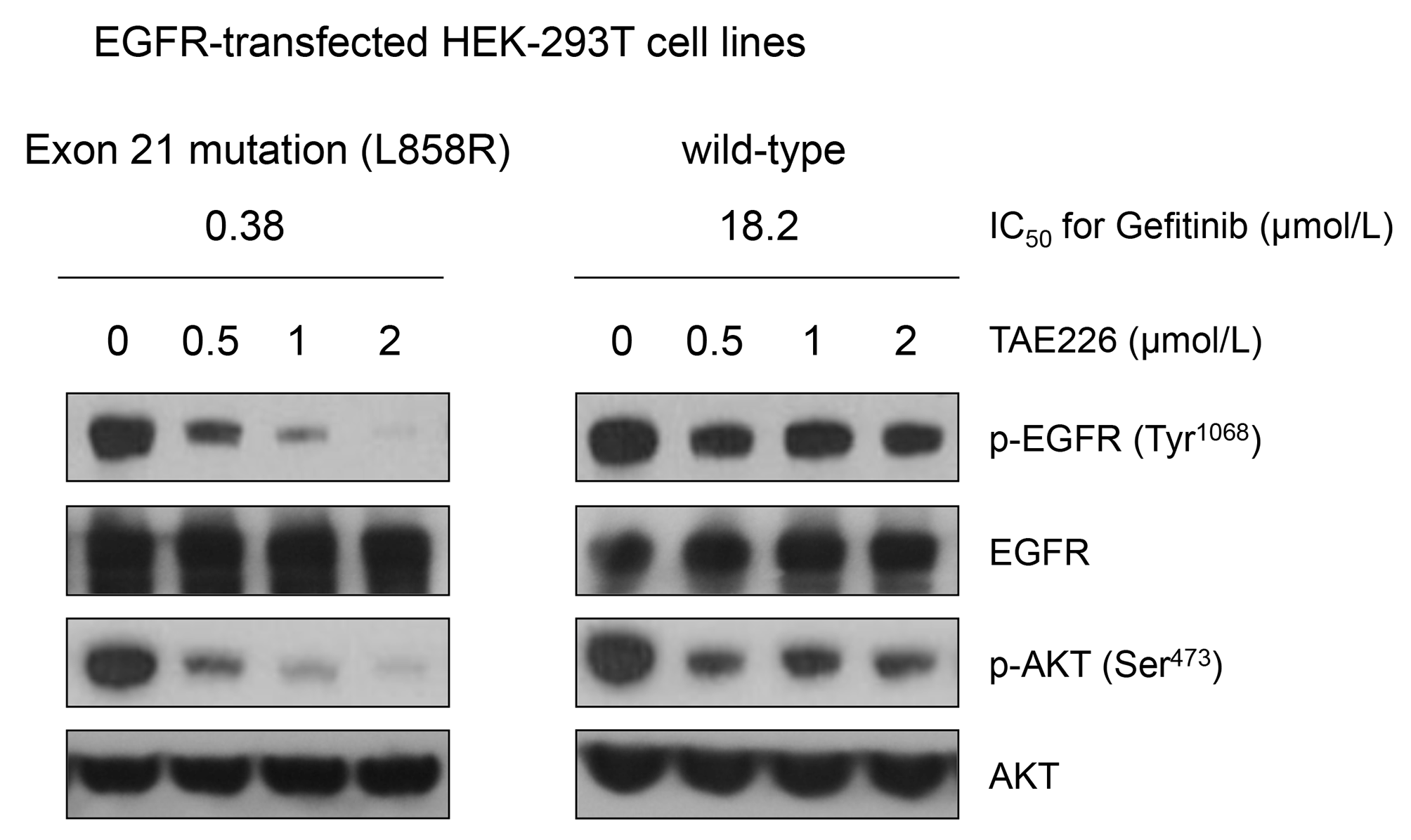

Supplement: S3 Fig — The mutant EGFR-transfected HEK-293T was more sensitive to gefitinib treatment than wild-type EGFR-transfected HEK-293T. TAE226 significantly inhibited phosphorylations of EGFR and AKT in mutant EGFR-trasnsfected HEK-293T compared with wild-type EGFR-transfected HEK-293T. (TIF) [file pone.0129838.s003.tif]

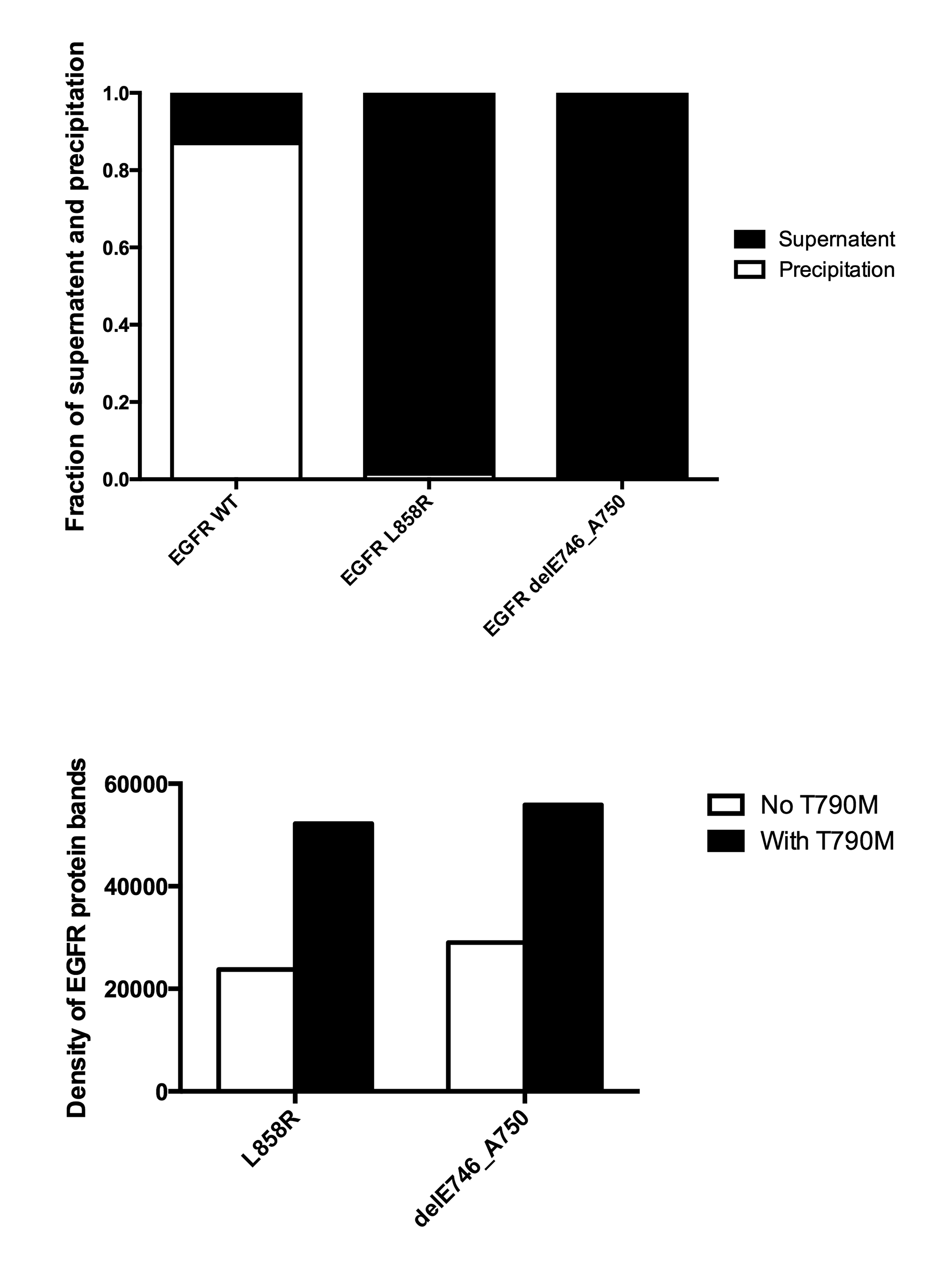

Supplement: S4 Fig — (A) Density of EGFR protein bands between the supernatant fraction and the precipitated fraction at 0.1 μM of TAE226 in Fig 2A was semi-quantified by densitometry analysis. The binding affinity of TAE226 to EGFR variants was approximately 7.5-fold higher to both mutant EGFRs than to wild-type EGFR. (B) Density of EGFR protein bands in supernatant fraction for TAE226 in Fig 2B was semi-quantified by densitometry analysis. Band density of EGFR protein for TAE226 was higher in T790M-containing EGFR mutant kinases (L858R/T790M or delE746_A750/T790M) than in common EGFR mutant kinases (L858R or delE746_A750). (TIF) [file pone.0129838.s004.tif]
