# Supplementary material for: TAE226, a Bis-Anilino Pyrimidine Compound, Inhibits the EGFR-Mutant Kinase Including T790M Mutant to Show Anti-Tumor Effect on EGFR-Mutant Non-Small Cell Lung Cancer Cells
Source: PLoS One. 2015 Jun 19;10(6):e0129838. doi: 10.1371/journal.pone.0129838 (PMC4474554; doi:10.1371/journal.pone.0129838)
Supplement: S1 Method — Detailed methods are described. (DOCX) [file pone.0129838.s005.docx]

**Otani et al.**

**TAE226, a bis-anilino pyrimidine compound, inhibits the EGFR-mutant kinase including T790M mutant to show anti-tumor effect on *EGFR*-mutant cells**

**S1 Method**

***In vitro* binding assay of the variant EGFR-TK domains**

Plasmid vectors expressing variant TK domains [wild-type (WT): 712-979 aa, exon 21 L858R mutation (L858R): 712-979 aa, and exon 19 deletion (ex19 del): 712-974 aa] of EGFR were constructed as Myc-HA-Flag-6His-tagged form at C-terminal end in each using the pDNR-CMV vector (Clontech, Carlsbad, CA). The variants (WT, L858R, and ex19 del) were corresponding to the partial cytoplasmic portions of EGFR mutants [wild-type, point mutation in exon 21 at codon 858 (L858R), and 15 base pairs in-frame deletion in exon 19 (ex19 del)], respectively. For in vitro binding assay, variant recombinant proteins for EGFR kinase domains (WT, L858R and ex19 del) were collected by immunoprecipitation method using anti-HA antibody linked agarose beads (Sigma-Aldrich) from HEK-293T cell lysates after 48 hours transfection with either WT, L858R or ex19 del expression constructs. At first, aliquots of the reconstituted variant proteins were incubated with ATP beads (ATP immorbilized on polyacrylamide resin; Novagen, a brand of EMD Biosciences, Inc., Darmstadt, Germany) for 2 hours at 4°C. After washing the beads, 10-1000 μM of ATP (Sigma-Aldrich) or 0.005-0.1 μM of TAE226 (Novartis Pharma AG) were added to the washed beads and further incubated for 10 min at room temperature. The beads-unbound supernatant and beads-bound precipitation fractions were collected separately. After addition of the SDS-PAGE sample buffer to the collected fractions, the samples were analyzed on SDS polyacrylamide gels, and western blots were done using indicated antibody. Bands in the western blots were semi-quantified using ImageJ software (National Institutes of Health, Bethesda, MD) and binding affinity of TAE226 to variant EGFR TK domains in the presence of ATP was estimated.

**Kinetic interaction analysis of TAE226 and gefitinib against wild-type EGFR and L858R/ T790M EGFR mutant**

To characterize the mechanism of the action of TAE226 on EGFR, we performed kinetic interaction analysis of TAE226 and gefitinib (as a control) against wild-type EGFR and L858R/T790M EGFR mutant by Proteros Reporter Displacement Assay (Proteros biostructures GmbH, Munich, Germany), which was used to determine the following parameters: K_d_, *k*_on_, *k*_off_ and residence time. The Proteros Reporter Displacement Assay was performed as described previously [1, 2].

In brief, the Proteros Reporter Displacement Assay is based on reporter probes that are designed to bind to the site of interest of the target protein. The proximity between reporter and protein results in the emission of an optical signal. Compounds that bind to the same site as the reporter probe displace the probe, causing signal diminution. Reporter displacement is measured over time after addition of compounds at various concentrations. In order to ensure that the rate of probe displacement reflects compound binding and not probe dissociation, probes are designed to have fast dissociation rates. Thus, compound binding and not probe dissociation is the rate-limiting step of probe displacement.

For K_d_ determination, percent probe displacement values are calculated for the last time point, at which the system has reached equilibrium. For each compound concentration, percent probe displacement values are calculated and plotted against the compound concentration. IC_50_-like values (corresponding to 50% probe displacement) are calculated using standard fitting algorithms. The reporter probe is used at a concentration reflecting its own K_d_ (probe) value. Thus, according to the Cheng Prusoff equation, the K_d_ value can be calculated with K_d_ = 1/2 IC_50_.

For determination of binding kinetics (*k*_on_, *k*_off_, and residence time), reporter displacement is plotted against time and fitted to a mono-exponential decay equation (probe binding = B + A x exp(-*k*_obs_ x t)) for each compound concentration. The resulting exponential coefficients equal the apparent association rate *k*_obs_. In a secondary plot, the *k*_obs_ values are plotted against the corresponding compound concentrations. Based on the equation *k*_obs_ = *k*_off_ + *k*_on_ x [compound], *k*_on_ values are extracted from the slope after linear fitting. The *k*_off_ values are calculated by K_d_ (compound) x *k*_on_. Residence time of the compound on the protein is calculated by 1/*k*_off_. For the secondary plot, only data are used from compound concentrations at which the *k*_obs_ values can be clearly determined. Data were omitted from the secondary plot analysis if (1) probe displacement was too fast to calculate accurate *k*_obs_ values, (2) compound concentration was too low to displace the probe significantly or (3) compound binding was faster than probe dissociation.

For wild-type EGFR and L858R/T790M EGFR mutant, the reporter probes used in this assay were based on a type I small molecule inhibitor. In order to normalize the binding affinity of the probes, each probe was used at an assay concentration that equals their K_d_ values. Namely, reporter probe concentrations for wild-type EGFR (50 nM) and L858R/T790M EGFR mutant (20 nM) were 215 nM (PRO-26), and 337 nM (PRO-15), respectively. Note that RPO-26 and RPO-15 are just the names of the probes from the manufacturer’s toolbox collection.

**References**

1. Schneider EV, Bottcher J, Huber R, Maskos K, Neumann L (2013) Structure-kinetic relationship study of CDK8/CycC specific compounds. Proc Natl Acad Sci U S A 110: 8081-8086.

2. Neumann L, von Konig K, Ullmann D (2011) HTS reporter displacement assay for fragment screening and fragment evolution toward leads with optimized binding kinetics, binding selectivity, and thermodynamic signature. Methods Enzymol 493: 299-320.
