# Supplementary material for: TAE226, a Bis-Anilino Pyrimidine Compound, Inhibits the EGFR-Mutant Kinase Including T790M Mutant to Show Anti-Tumor Effect on EGFR-Mutant Non-Small Cell Lung Cancer Cells
Source: PLoS One. 2015 Jun 19;10(6):e0129838. doi: 10.1371/journal.pone.0129838 (PMC4474554; doi:10.1371/journal.pone.0129838)
Supplement: S1 Table — This table is updated data from ref. 20. (DOCX) [file pone.0129838.s006.docx]

**S1 Table.** *In vitro* non-cellular kinase assay for TAE226

| Kinase | IC_50_ (µM) |
| --- | --- |
| FAK | 0.0055 |
| IGF-1R | 0.14 |
| Pyk2 | 0.04 |
| Insulin-R | 0.044 |
| c-Src | 0.91 |
| Syk | 7.9 |
| Lck | > 10 |
| ZAP-70 | 7.7 |
| EGFR | 1.7 |
| HER2 | 0.95 |
| KDR | 0.36 |
| Flt-1 | 3.4 |
| Flt-3 | 0.48 |
| Flt-4 | 0.22 |
| FGFR-1 | 0.75 |
| FGFR-3 (K650E) | 0.63 |
| c-Met | 0.16 |
| c-Abl | 5.9 |
| c-Kit | > 10 |
| PDGFR | 2.6 |
| Tek | 0.58 |
| Ret | 1.3 |
| Ret (M918T) | 2.3 |
| JAK-2 | 0.84 |
| ALK | 0.15 |
| CDK1/cyclin B | 0.54 |
| c-Raf | > 10 |
| BRAF (V600E) | 1.4 |
| PDK1 | > 10 |
| PKA | > 10 |
| PKB/AKT | > 10 |
