# Supplementary material for: TAE226, a Bis-Anilino Pyrimidine Compound, Inhibits the EGFR-Mutant Kinase Including T790M Mutant to Show Anti-Tumor Effect on EGFR-Mutant Non-Small Cell Lung Cancer Cells
Source: PLoS One. 2015 Jun 19;10(6):e0129838. doi: 10.1371/journal.pone.0129838 (PMC4474554; doi:10.1371/journal.pone.0129838)
Supplement: S2 Table — Mean IC50 and SD were calculated using the value of each IC50. (DOCX) [file pone.0129838.s007.docx]

| **S2 Table.** IC_50_ values in independent assays and calculated SD for TAE226 and Gefitinib in each cell line | | | | | | | | | | | | | | | | |
| --- | --- | --- | --- | --- | --- | --- | --- | --- | --- | --- | --- | --- | --- | --- | --- | --- |
| Cell lines | IC_50_ and SD for TAE226 | | | | | | | | | IC_50_ and SD for Gefitinib | | | | | | |
|  | each IC_50_ | | | | | | mean IC_50_ | SD | *n* | each IC_50_ | | | | mean IC_50_ | SD | *n* |
| PC-9 | 0.19 | 0.16 | 0.15 | 0.16 | 0.14 | 0.14 | 0.16 | 0.01 | 6 | 0.0020 | 0.0020 | 0.0031 | 0.0039 | 0.0028 | 0.0008 | 4 |
| HCC827 | 0.061 | 0.095 | 0.090 | 0.10 |  |  | 0.086 | 0.01 | 4 | 0.0014 | 0.0015 | 0.0014 | 0.0015 | 0.0014 | 0.00005 | 4 |
| NCI-H3255 | 0.12 | 0.12 | 0.13 | 0.12 |  |  | 0.12 | 0.005 | 4 | 0.0016 | 0.0017 | 0.0015 | 0.0020 | 0.0017 | 0.0002 | 4 |
| RPC-9 | 0.35 | 0.33 | 0.28 | 0.27 |  |  | 0.31 | 0.03 | 4 | 12.0 | 11.4 | 10.1 | 9.73 | 10.8 | 0.93 | 4 |
| NCI-H1975 | 0.16 | 0.20 | 0.15 | 0.19 |  |  | 0.17 | 0.02 | 4 | 7.4 | 7.8 | 7.0 | 7.4 | 7.4 | 0.29 | 4 |
| NCI-H2228 | 0.28 | 0.28 |  |  |  |  | 0.28 | 0.001 | 2 | 12.4 | 11.1 |  |  | 11.7 | 0.64 | 2 |
| NCI-H1666 | 0.50 | 0.34 |  |  |  |  | 0.42 | 0.08 | 2 | 11.1 | 12.7 |  |  | 11.9 | 0.80 | 2 |
| NCI-H1395 | 0.48 | 0.48 |  |  |  |  | 0.48 | 0.002 | 2 | ND | | | | ND | ND | ND |
| A549 | 1.4 | 1.4 | 1.4 | 1.3 |  |  | 1.4 | 0.05 | 4 | 21.0 | 23.4 | 20.9 | 21.0 | 21.6 | 1.0 | 4 |
| NCI-H1648 | 1.7 | 1.8 |  |  |  |  | 1.7 | 0.07 | 2 | 8.1 | 9.4 |  |  | 8.7 | 0.65 | 2 |
| NCI-H1819 | 4.0 | 3.2 | 4.0 | 4.1 |  |  | 3.8 | 0.4 | 4 | 6.7 | 6.6 |  |  | 6.6 | 0.059 | 2 |
| Calu-3 | 4.5 | 3.8 |  |  |  |  | 4.1 | 0.37 | 2 | 5.9 | 4.6 |  |  | 5.3 | 0.64 | 2 |
| SK-BR-3 | 34.6 | 35.6 |  |  |  |  | 35.1 | 0.53 | 2 | ND | | | | ND | ND | ND |
| NCI-H1993 | 0.89 | 0.89 |  |  |  |  | 0.89 | 0.001 | 2 | 12.2 | 12.9 |  |  | 12.5 | 0.36 | 2 |
| MKN45 | 1.04 | 1.16 |  |  |  |  | 1.10 | 0.06 | 2 | ND | | | | ND | ND | ND |
| NCI-H838 | 6.3 | 6.2 |  |  |  |  | 6.2 | 0.02 | 2 | 28.3 | 29.0 |  |  | 28.6 | 0.35 | 2 |
| NCI-H1299 | 3.1 | 3.0 | 2.7 | 2.4 |  |  | 2.8 | 0.26 | 4 | 31.7 | 34.6 | 30.5 | 33.6 | 32.6 | 1.6 | 4 |
| HEK293T-L858R | 0.50 | 0.36 | 0.43 | 0.34 |  |  | 0.41 | 0.06 | 4 | 0.46 | 0.28 | 0.48 | 0.31 | 0.38 | 0.087 | 4 |
| HEK293T-WT EGFR | 3.0 | 2.8 | 3.1 | 3.0 |  |  | 3.0 | 0.11 | 4 | 12.4 | 22.6 | 17.3 | 20.7 | 18.2 | 3.9 | 4 |
| Mean IC_50_ and SD were calculated using the value of each IC_50_. IC_50_, inhibitory concentration at 50%; SD, standard deviation; *n*, the number of MTS assays performed for each cell line; ND, not done | | | | | | | | | | | | | | | | |
